# An Automatic Random Walker Algorithm for Segmentation of Ground Glass Opacity Pulmonary Nodules

**DOI:** 10.1155/2022/6727957

**Published:** 2022-09-29

**Authors:** Xiangxia Li, Bin Li, Hua Yin, Bo Xu

**Affiliations:** ^1^School of Information Engineering, Guangdong University of Finance & Economics, Guangzhou, Guangdong, China; ^2^School of Automation Science and Engineering, South China University of Technology, Guangzhou, China

## Abstract

Automatic and accurate segmentation of ground glass opacity (GGO) nodules still remains challenging due to inhomogeneous interiors, irregular shapes, and blurred boundaries from different patients. Despite successful applications in the image processing domains, the random walk has some limitations for segmentation of GGO pulmonary nodules. In this paper, an improved random walker method is proposed for the segmentation of GGO nodules. To calculate a new affinity matrix, intensity, spatial, and texture features are incorporated. It strengthens discriminative power between two adjacent nodes on the graph. To address the problem of robustness in seed acquisition, the geodesic distance is introduced and a novel local search strategy is presented to automatically acquire reliable seeds. For segmentation, a label constraint term is introduced to the energy function of original random walker, which alleviates the accumulation of errors caused by the initial seeds acquisition. Massive experiments conducted on Lung Images Dataset Consortium (LIDC) demonstrate that the proposed method achieves visually satisfactory results without user interactions. Both qualitative and quantitative evaluations also demonstrate that the proposed method obtains better performance compared with conventional random walker method and state-of-the-art segmentation methods in terms of the overlap score and F-measure.

## 1. Introduction

Lung cancer is the leading cause of cancer-related deaths among both men and women. Lung cancer accounts for approximately 27% of all cancer deaths because its diagnosis occurs at the advanced stages of the disease. Lung cancer is controllable if it is timely diagnosed and appropriately treated in an early stage. Currently, only 15% of all lung cancers are diagnosed at an early stage, which causes a five-year survival rate of only 16%. Therefore, lung cancer diagnosis is of importance to increase the chances of survival and reduce the mortality rate in an early stage, when treatment options are better. Lung cancer potentially manifests itself as pulmonary nodules [1]. Computed tomography (CT) is one of the most prevalent modalities for early inspection and analysis of pulmonary nodules. In recent years, medical image processing research has been underway for detection and segmentation of pulmonary nodules in CT images. In particular, segmentation of pulmonary nodules is a worthy task for subsequent planning of treatment strategies, monitoring of disease progression, and prediction of treatment outcome, because some key indicators can be readily calculated in segmented pulmonary nodules, such as volume [2, 3] and size [4]. In clinical routine, segmentation of pulmonary nodules is manually delineated in a slice-by-slice manner under the guidance of radiologists. However, manual segmentation is time-consuming and subjective in larger studies. Therefore, automatic segmentation of pulmonary nodules is highly desirable to relieve radiologist workload.

Based on position of pulmonary nodules in the lung parenchyma and proximity to other anatomical structures, five types of nodules are identified: well-circumscribed, juxta-vascular, juxta-pleural, cavitary pulmonary nodules, and ground glass opacities (GGO). Over the last decades, a lot of efforts have been devoted to studying the segmentation of pulmonary nodules. However, most of nodule segmentation methods that have been previously published focus on well-circumscribed pulmonary nodule segmentation. There have been limited literature on segmentation of GGO pulmonary nodules. Unfortunately, GGO pulmonary nodules have a higher malignancy rate than other solitary nodules [5]. Therefore, an accurate and efficient method is urgently demanded for segmentation of GGO nodules, which is the focus of our paper.

Segmentation of GGO pulmonary nodules still remains challenging due to inhomogeneous interiors, blurred boundaries, and irregular shapes from different patients. In this paper, an improved random walker-based method is proposed for segmentation of GGO pulmonary nodules. The random walker was firstly introduced by Grady [6] for interactive image segmentation. The initialization of random walker model requires user inputs to guide the segmentation process. The user provides some seeds that indicate certain pixels as the object itself and few others as the background. For each pixel, it is necessary to compute the probability that a random walker leaving the pixel will first arrive at each seed. Many models of random walker have been widely applied in many image segmentation processing tasks [7–9].

Random walker model needs the user to specify object and background seed points. However, it is extremely sensitive to the locations and quantity of seeds. Once the locations of seeds are not precise and the quantity of seeds is not sufficient for accurate pulmonary nodule segmentation, unlabeled pixels can be assigned the wrong labels. This may lead to degrading the quality of segmentation. Therefore, the user needs frequently select seed points that could improve the segmentation performance. However, this process is considerably tedious and the computational cost is extremely expensive. In addition, user interaction seriously restricts the applications of random walker method. In clinical practices, most clinicians would benefit from automated methods. This motivates us to present an alternative solution strategy for automatic seeds acquisition. Please see Section 3.1 for more details.

In addition, the random walker method might be prone to get stuck at a local minimum in the energy landscape. At present, some existing random walker methods have attempted to exploit some prior knowledge to address this shortcoming. For instance, T. Messay et al. [10] proposed the guided random walk method for left ventricle segmentation. The authors incorporated prior knowledge into the energy function of the random walks. However, the sensitivity of seeds location and quantity has not been addressed under guided random walk framework. Mi et al. [11] proposed an iterative method based on random walkers for tumor segmentation. The authors took into account prior knowledge of the influence of tumor growth prediction. In this paper, an improved random walker method is proposed for segmentation of pulmonary nodules. A constraint term is introduced by extending the fundamental energy function of the random walker. The main contributions of the proposed method are summarized as follows:

1. Introducing an alternative solution strategy for automatic seeds acquisition. Different from many random walker methods, we propose a fully automatic method for segmentation of GGO pulmonary nodules. An automatic solution strategy is proposed to locate nodule and background seeds. Subsequently, the acquired seeds are further fed to the random walker.
2. Constructing a new affinity matrix that measures the similarity between a pair of neighboring nodes in some predefined feature space. The affinity entry is composed of two components: an adjacency component and a feature-based component. The adjacency component is defined based on the spatial distance to enforce the spatial coherence. The feature-based component is defined based on intensity and texture features. Finally, two components are combined by a point-wise multiplication operation.
3. Defining a novel energy function for segmentation of GGO nodules. A label constraint term is introduced to the fundamental energy function of the random walker, which alleviates the accumulation of errors caused by the acquired seeds.

The remainder of the paper is organized as follows.  Section 2 briefly reviews the relevant literature for segmentation of pulmonary nodules. The details of the proposed method are described in Section 3. Extensive experimental evaluations are conducted on the LIDC dataset and experimental results are given, which show excellent performance of the proposed method in comparison with traditional random walker and several other previously published methods. The overlap score, F-measure, and executive time are discussed in Section 4. Discussions are given in Section 5. Finally, conclusions and future work are provided in Section 6.

## 2. Related Works

Over the past decade, a lot of efforts have been devoted to the segmentation of pulmonary nodules. These methods can be broadly categorized as either intensity-based or shape-based and deep learning methods. The former separates pulmonary nodules from the surrounding background by using purely intensity information, such as thresholding [12, 13], pixels classification [14], region growing [15, 16], clustering [17–19], and mathematical morphology [20, 21]. Some hybrid methods have been applied in pulmonary nodule segmentation. Dehmeshi et al. [16] incorporated the thresholding and region growing methods for the segmentation of the pulmonary nodules. The mathematical morphology-based methods also have been applied to segmentation of pulmonary nodules. The main difficulty with these methods is to decide a suitable size of structuring elements to segment all different kinds of pulmonary nodules. Shape-based methods [22, 23] for segmentation of pulmonary nodules have been well studied. This category yields better segmentation results than the former, since it takes into account the nodule-specific geometrical constraints for segmentation of pulmonary nodules. Strictly speaking, these geometrical assumptions are not valid by ground glass opacity (GGO) nodules because these types of pulmonary nodules show the large variability of the morphology.

Deformable models [24–27] have been attracting more and more attention for segmentation of pulmonary nodules. Deformable models are flexible to cope with topological variability. They have been shown to achieve very good segmentation results. Although these deformable models have achieved satisfactory segmentation of pulmonary nodules with strong boundaries, they are highly sensitive to noise and are also dependent on the location of the initial contour. Meanwhile, deformable models demand the high computational burden due to the use of a huge number of iterations.

Deep learning techniques [28–35] have been used extensively in segmentation of pulmonary nodules. Compared with traditional segmentation methods, deep learning uses the deep neural network models to train a large number of images. According to the data, deep learning actively learns the low-level features of nodules and forms more abstract high-level features, so as to achieve a better segmentation. Although these deep learning models have achieved satisfactory segmentation of pulmonary nodules, deep learning needs the setting of hyperparameters in the network model, and the challenges are still not very well understood. Different from these models, our proposed method performs simply and efficiently and obtains the segmentation results by solving a linear system. In addition, the proposed method is more robust against intensity variations and noise than deformable models, since the random walkers capture the spatial connectivity much better than deformable models. The details of the proposed method are described in the following section.

## 3. Method

**Figure 1:**
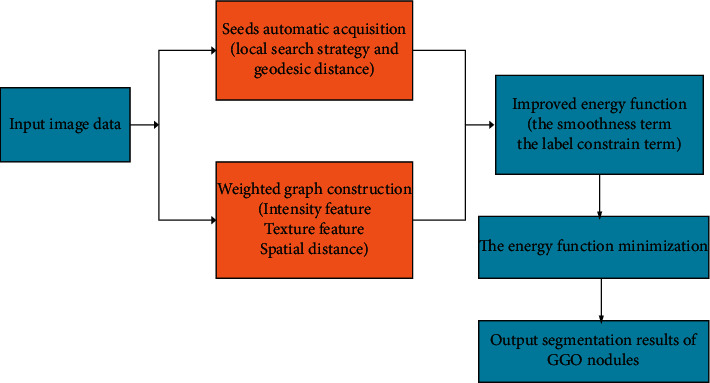
The flowchart of the improved random walker for GGO nodule segmentation.

In this section, an improved random walker method is proposed for segmentation of GGO pulmonary nodules, which consists of four main steps: acquisition of seeds, construction of undirected weighted graph, designation of the energy function, and optimization of the energy function. The flowchart of the proposed method for GGO nodule segmentation is shown in Figure 1. The details of each step are described in the following sections. Before the improved random walker method is presented, the description of some basic notations is firstly given in this section.

### 3.1. Acquisition of Seeds

After segmenting the GGO pulmonary nodules by the provided random walk method, nodule and background seed points should be first acquired. In this section, an efficient local search strategy is presented for acquiring the reliable seeds, which is often unattainable for most of the existing studies of random walker methods.

**Figure 2:**
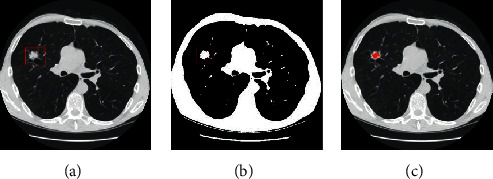
Acquisition of nodule seeds. (a) A GGO nodule in a coherence filtered lung image, highlighted by a red rectangle. (b) Initial segmentation *O* by adaptive thresholding method. (c) A binary region *O* by morphological open operation and connection component analysis.

**Figure 3:**
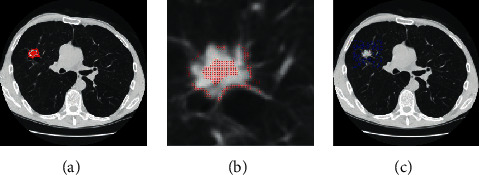
Acquisition of nodule and background seeds. (a) The final nodule seeds by using a local search strategy, marked by red spots. (b) An enlarged mask of nodule seeds. (c) The background seeds by using local search strategy, marked by blue spots.

The preprocessing stage is essential within a lung CT image before pulmonary nodules segmentation. The coherence filter is adopted to remove the effect of the image noise while preserving the nodule boundaries very well. The random walk segmentation method strongly depends on the initial seed acquisition. The user needs to frequently acquire seed points until the satisfactory segmentation results are achieved. However, the process is considerably tedious and time-consuming for large-scale images. Therefore, automatic acquisition of seed points is essential for the next step. We will focus on presenting an effective method to make the manual seed acquisition automatically which reduces user interaction. Firstly, the adaptive threshold method [12] is adopted to find a global threshold. The pixels are roughly identified as a part of pulmonary nodule, whose intensities are greater than the global threshold. The remaining pixels are considered as the background pixels. Figures 2 and 3 show an example of the nodule and background seeds acquired using the proposed method as red and blue spots, respectively.  Figure 2(b) shows the binary image in the filtered CT image. As observed from Figure 2(b), the adaptive threshold can roughly separate the nodule from the background. The morphological open operation is used to eliminate small holes and noises and connection component analysis is employed to remove the undesired regions. Finally, an initial segmentation result *O* is obtained.

Secondly, a small region *O*_0_ is created based on geodesic distance [36] from the initial segmentation result *O*. *O*_0_ is the largest connected component within *M* and *M* − *T*, where *M* is the maximum geodesic distance value starting from the boundary *•O* to the center of *O* and *T* is a predefined parameter. The pixels in the *O*_0_ region are identified as the nodule seeds. Noticeably, nodule seeds may be restricted to a homogeneous part of the nodule.  Figure 2(c) shows the nodule seeds marked with red spots. However, it is also well known that GGO pulmonary nodules are often inhomogeneous. The accuracy of GGO nodule seed acquisition could be further improved if more nodule seeds are available. We need to acquire the nodule seeds as uniformly as possible for accurate segmentation of GGO pulmonary nodules. Hence, we introduce a local search strategy to acquire the other nodule seeds. The pixels of *•O* are used as the initial pixels to search the other nodule seeds. If pixels are close to the boundary *•O* of *O* and have features similar to those of pixels in the *O*_0_ region, they will be identified as the nodule seeds.

In GGO pulmonary nodules with intensity inhomogeneity, the use of intensity feature alone will not be sufficient. Texture feature provides complementary information of intensity feature. Texture feature gives a measure of the variation in the intensities at pixels of interest, which has been proved to be extremely effective for pulmonary nodule detection [37–40]. For calculating texture feature, gray-level cooccurrence matrix (GLCM) [40] and Gabor filter are used in this paper. The GLCM is generated by counting the occurrences of intensity pairs between the current and neighbor pixels of *l*-gray-level image. The normalized GLCM is calculated in the following equation:

(1) $$\begin{array}{c} P\left(i,j\right)=\frac{N\left(i,j\right)}{\sum_{m=0}^{l-1}\sum_{n=0}^{l-1}N\left(m,n\right)}, \end{array}$$

where *i* and *j* are intensity values in the *l*-gray level. *N*(*i*, *j*) is the relative frequency matrix given in the following equation:

(2) $$\begin{array}{c} N\left(i,j\right)=\text{num}\left\{\begin{array}{c} \left(\left(m_{x},m_{y}\right),\left(n_{x},n_{y}\right)\in \left(V_{x}\times V_{y}\right)\times \left(V_{x}\times V_{y}\right)\right)\\ \max\left(\left|m_{x}-n_{x}\right|,\left|m_{y}-n_{y}\right|\right)\leq d\\ I_{l-\text{gray}}\left(m_{x},m_{y}\right)=i,I_{l-\text{gray}}\left(n_{x},n_{y}\right)=j \end{array}\right\}, \end{array}$$

where *V*_*x*_ and *V*_*y*_ are the *x*-axis and *y*-axis spatial domains, respectively. (*m*_*x*_, *m*_*y*_) and (*n*_*x*_, *n*_*y*_) are pixel positions. *I*_*l*−gray_ is the *l*-gray level. Gabor transformation [41] is another commonly used texture feature extraction method. A Gabor filter is the multiplication of a Gaussian distribution by a harmonic, which is formulated as follows:

(3) $$\begin{array}{c} \psi \left(x,y\right)=\exp\left(\frac{-x^{2}+\gamma y^{2}}{2\sigma^{2}}\right)\cdot \;\cos\left(2x\cdot \frac{x{}^{\prime }}{\lambda }+\varphi \right), \end{array}$$

(4) $$\begin{array}{c} x{}^{\prime }=x\;\cos\;\theta +y\;\sin\;\theta , \end{array}$$

where *σ* denotes the standard deviation of 2D Gaussian envelope. *λ* and *θ* are wavelength and orientation, respectively. *φ* and *γ* are phase shift and spatial aspect ratio, respectively. In this paper, eight orientations *θ* = 0, *π*/8,…, 7*π*/8, two wavelengths *λ* = 1,2, and two standard deviations *σ* = 1,2 are employed to extract texture features. The magnitude map of Gabor filter is calculated to describe the local texture features in this paper. The corresponding maximum amplitude is defined in the following equation:

(5) $$\begin{array}{c} I{}^{\prime }\left(x,y\right)=\underset{k}{\max}I_{k}^{G}\left(x,y\right), \end{array}$$

where *I*_*k*_^*G*^(*x*, *y*) is the filtered image by the set of Gabor filters. *k* is the number of Gabor filters with different orientation *θ*. After the intensity and texture features are extracted, a local search strategy is introduced to acquire the other nodule and background seeds. Let Ω be a boundary Lipschitz domain and let *I* : Ω ⊂ ℝ^2^⟶*ℐ* be defined as gray-level image function. The similarity between a pixel *i* in *•O* and its adjacent pixel *j* is calculated as follows:

(6) $$\begin{array}{c} S\left(i,j\right)={\left\|T_{i}-T_{j}\right\|}^{2}\times e^{{\left\|I_{i}-I_{j}\right\|}^{2}}j\in N_{i}, \end{array}$$

where *I*_*i*_ and *I*_*j*_ denote the intensity values at pixel *i* and its adjacent pixel *j*, respectively. Eight-neighbor connections at a pixel are used in this paper. *T*_*i*_ and *T*_*j*_ denote the texture values at pixels *i* and *j*, respectively. ‖·‖ denotes the *L*_2_ norm to measure the feature difference between two adjacent pixels. *N*_*i*_ is a neighborhood system of a pixel *i*. Herein, the exponential function is used to stress the importance of intensity feature. If the similarity satisfies [34] the rule in (7), we will add pixels to the nodule seed set *V*_*M*_^*F*^.

(7) $$\begin{array}{c} V_{M}^{F}=V_{M}^{F}\cup \left\{j\right\}\mathrm{if}\;S\left(i,j\right)<\kappa ,\\ S\left(j,k\right)<\kappa ,i\in \mathrm{•O},k\in O_{0},j\in N_{i}, \end{array}$$

where *κ* is a predefined threshold. We empirically set *κ* = 10, which works well in our method. The rule is very simple and effective. The local search strategy is iteratively implemented and the iteration stops when all pixels of *•O* undergo the boundary. The new identified nodule seeds are added to obtain the final nodule seeds set *V*_*M*_^*F*^.  Figure 3(a) shows the final nodule seeds marked with red spots.  Figure 3(b) shows the zoomed-in region of Figure 3(a).

After the nodule seeds are acquired, we will automatically acquire the background seeds. If pixels are close to the boundary *•O* of *O* and have a large feature difference with the pixels of the region *O*_0_, they will be identified as the background seeds. If the similarity satisfies the following rule, we will add pixels to the background seed set *V*_*M*_^*B*^. The rule is defined in (8).

*V*_*M*_^*B*^=*V*_*M*_^*B*^ ∪ {*j*, *j*′}if *S*(*i*, *j*) > *η* and *S*(*j*, *k*) > *η*, *i* ∈ *•O*  , *k* ∈ *O*_0_, *j* ∈ *O*_*b*_/*O*_0_.

*S*(*i*, *j*′) > *η* and *S*(*j*′, *k*) > *η*.

(8) $$\begin{array}{c} j{}^{\prime }\in N\left(j\right), \end{array}$$

where *O*_*b*_ is a region that the distance of two pixel positions from the center of the pulmonary nodule region to pixels outside the region *O* satisfies a predefined threshold *T*_1_. The background seed set *V*_*M*_^*B*^ will be updated and the iteration stops when there are no new pixels of the region *O*_*b*_ to be added to the background seed set *V*_*M*_^*B*^.  Figure 3(c) shows the background seeds marked with blue spots.

### 3.2. Undirected Weighted Graph Construction

The construction of the suitable graph is inevitable. The key step on the graph construction is to define a discriminative affinity matrix. An unreasonable affinity weight potentially captures erroneous spatial relationship between two adjacent pixels, resulting in accumulating the erroneous information. Further, errors are conveyed into the subsequent step of the energy function of random walker.

The input image *ℐ* is represented as an undirected weighted graph *G*=(*V*, *E*, *W*), where *V* is a set of nodes and *E* is a set of edges. The image consists of *n* pixels. A node *v*_*i*_ ∈ *V* represents the *i*^*th*^ pixel of the input image. An edge *e*_*ij*_ ∈ *E* connects a pair of neighboring nodes *v*_*i*_ and *v*_*j*_. Edges are weighted by the nonnegative weighted function *W*=[*w*_*ij*_], where *w*_*ij*_ : *E*⟶*IR*^+^ ∪ {0} on edge *e*_*ij*_ reflects the similarity between two neighboring nodes *v*_*i*_ and *v*_*j*_. Note that *w*_*ij*_=0 is considered nonrelevant relation between a pair of nodes *v*_*i*_ and *v*_*j*_. In addition, the edge weights are symmetric; that is, *w*_*ij*_=*w*_*ji*_ in an undirected graph. The nodule seed set *V*_*M*_^*F*^ along with the background seed set *V*_*M*_^*B*^ will constitute the overall seed set *V*_*M*_=*V*_*M*_^*F*^ ∪ *V*_*M*_^*B*^. The remaining unlabeled pixels are denoted as *V*_*U*_ ⊂ *V*, such that *V*_*M*_ ∪ *V*_*U*_=*V* and *V*_*M*_∩*V*_*U*_=*ϕ*.

### 3.3. Definition of an Affinity Matrix

The success of random walker depends on how accurately the relationships between two neighboring nodes of the weighted graph represent the pulmonary nodule and background. In other words, the nodes belonging to pulmonary nodules should have the high affinity among them. The nodes belonging to background should also have the high affinity among them. The key step of the graph construction is to define an affinity matrix *W*=[*w*_*ij*_], which measures the similarity between two neighboring nodes in some predefined feature space. Apparently, intensity feature alone is insufficient for segmentation of GGO pulmonary nodules. In general, different features describe an object characteristic from different views and provide complementary information to each other, such as texture feature, Haar feature, and histogram of gradient (HOG) feature. We have managed to exploit texture feature and spatial distance to define an affinity matrix. Texture descriptor can be a characterized property of object surface, such as contrast, regularity, coarseness, and structural arrangement. Recently, the efficiency of texture feature has been proved [43, 44]. Distinct from [43, 44], we integrate local binary pattern (LBP) [45–47] and Gabor filter [48] to extract texture features. In addition, the spatial distance is employed to control the spatial influence between two adjacent nodes. In other words, the closer two nodes are in spatial distance, the more likely they are to influence each other.

The affinity entry between a pixel and its neighbors is calculated by incorporating texture feature and spatial distance. It is composed of two components: an adjacency component and a feature-based component. The adjacency component is defined based on the spatial distance. The closer two nodes are, the stronger the penalty imposing the similar labels is. In other words, increasing the spatial distance will decrease the affinity entry. The feature-based component is defined based on intensity and texture features. We adopt Gaussian kernel function with affinity measurement for simplicity. Euclidean distances of features between two neighboring nodes are associated with the edges of the weighted graph. Consequently, two components are combined by a point-wise multiplication operation. Hence, the affinity entry *w*_*ij*_ from a node *i* to its neighboring node *j* is calculated in the following equation:

(9) $$\begin{array}{c} w_{ij}=\exp\left(-{\left\|z_{i}-z_{j}\right\|}_{2}\right)\exp\left(-{\left\|I\left(i\right)-I\left(j\right)\right\|}_{2}\right)\cdot \;\exp\left(-{\left\|T\left(i\right)-T\left(j\right)\right\|}_{2}\right), \end{array}$$

where *z*_*i*_ and *z*_*j*_ denote the spatial coordinates of nodes *i* and *j*, respectively. ‖ . ‖_2_ denotes the *L*_2_ norm to measure the distance between a pair of adjacent nodes on each feature space. The higher affinity entry *w*_*ij*_ indicates the higher discriminative power of the representative features. As a result, they are assigned the same label. Meanwhile, the smaller affinity entry *w*_*ij*_ indicates that feature differences between two neighboring nodes will be the larger; thus the guided random walker tends to cross these edges. With the help of the discriminative affinity matrix, it can make segmentation results of pulmonary nodules more accurate and efficient. After the affinity matrix is constructed, we will discuss how to design a new energy function for accurate GGO pulmonary nodule segmentation, which is described in more details in Section 3.3.

### 3.4. The Energy Function Designation

The random walker segmentation method is formulated as an energy function minimization problem. The fundamental energy function of the random walk imposes the consistency in the labels of neighboring pairs. In other words, the labels will be assigned the same in a neighborhood system, when their corresponding features are similar. How to adequately use the acquired seeds is crucial for accurate segmentation of GGO pulmonary nodules. Note that it is not always possible to accurately initialize seeds from the segmented pulmonary nodule regions. Once the initial segmentation results are not precise, the label assignment inevitably shows the potential errors. The accumulation of errors can degrade the quality of segmentation. To address this problem, a label constraint term is added to the fundamental energy function of the random walk by incorporating the prior knowledge of seeds. Then, a weight function of the label constraint term is based on fuzzy membership value, which is discussed below.

The fuzzy membership is the degree of membership of a node belonging to the foreground or background. In this paper, we build a foreground Gaussian Mixture Model (denoted as *GMM*^*F*^) and a background Gaussian Mixture Model (denoted as *GMM*^*B*^) as the global guidance for segmentation of GGO pulmonary nodules, where Gaussian Mixture Model (GMM) is generated from the acquired seeds. Fuzzy membership value is calculated based on the posterior probability of Gaussian Mixture Model (GMM). The intensity and texture features are incorporated to construct an augmented feature vector *h*=[*I*, *T*]. An indexing function *ℱ* : Ω⟶*ℒ* is defined, where *ℒ* indicates that the probabilities of the nodes are assigned a node *i*. *ℱ*(*i*)=1 indicates the assignment of a node *i* to the foreground and *ℱ*(*i*)=−1 indicates the assignment of a node *i* to the background. *P*_*GMM*_(*h*(*i*), 1) represents the posterior probability of a node *i* to belong to nodules and *P*_*GMM*_(*h*(*i*), −1) represents the posterior probability function of a node *i* to belong to background. The higher value *P*_*GMM*_(*h*(*i*), 1) has, the higher the probability that node *i* belongs to *V*_*M*_^*F*^. A similar expression is applicable to *P*_*GMM*_(*h*(*i*), −1). The weight function will be assigned to a large value when the predefined label and the calculated label are similar during the energy minimization. To obtain a desired probability vector *ℱ*=[*ℱ*(*i*)]_*N*×1_, the energy function is defined in the following equation:

(10) $$\begin{array}{c} E\left(ℱ\right)=\frac{1}{2}{\sum_{e_{ij}\in E}w_{ij}\left(ℱ\left(i\right)-ℱ\left(j\right)\right)}^{2}+\frac{1}{2}\alpha \left(\sum_{i=1}^{\left|V_{M}\right|}u_{i}{\left(ℱ\left(i\right)-b\left(i\right)\right)}^{2}\right), \end{array}$$

where *ℱ*(*i*) and *ℱ*(*j*) represent the probabilities on nodes *i* and *j* and *u*_*i*_ is the membership function on a node *i*. *α* is a tradeoff parameter and |*V*_*M*_| is the number of seeds. *b*(*i*) is the preassigned label on a node *i*, which is defined as follows:

(11) $$\begin{array}{c} b\left(i\right)=\left\{\begin{array}{c} -1,\;if\;i\in V_{M}^{B},\\ 1,\;if\;i\in V_{M}^{F}, \end{array}\right. \end{array}$$

and *u*_*i*_ is calculated as follows:

(12) $$\begin{array}{c} u_{i}=e^{-\log\left(P_{GMM}\left(h\left(i\right),b\left(i\right)\right)\right)/\log\left(P_{GMM}\left(h\left(i\right),1\right)\right)+\log\left(P_{GMM}\left(h\left(i\right),-1\right)\right)}. \end{array}$$

The label constraint term enforces the consistency between the calculated probability and the preassigned probability after the energy minimization, which reflects the information of seeds. After the energy function is defined, we will discuss the minimization of the energy function in Section 3.4.

### 3.5. The Energy Function Minimization

The energy function is minimized by expanding (10) and (13) is in the matrix form.

(13) $$\begin{array}{c} E\left(ℱ\right)=\frac{1}{2}{\sum_{e_{ij}\in E}w_{ij}\left(ℱ\left(i\right)-ℱ\left(j\right)\right)}^{2}+\frac{1}{2}\alpha \left(\sum_{i=1}^{\left|V_{M}\right|}u_{i}{\left(ℱ\left(i\right)-b\left(i\right)\right)}^{2}\right)\\ =\frac{1}{2}\sum_{i,j\in N}w_{ij}\left(ℱ{\left(i\right)}^{2}-2ℱ\left(i\right)ℱ\left(j\right)+ℱ{\left(j\right)}^{2}\right)\\ +\frac{1}{2}\alpha \sum_{i=1}^{\left|V_{M}\right|}u_{i}\left(ℱ{\left(i\right)}^{2}-2ℱ\left(i\right)b\left(i\right)+b{\left(i\right)}^{2}\right)\\ =\frac{1}{2}{ℱ}^{T}\left(D-W\right)ℱ+\frac{1}{2}\alpha {\left(ℱ-b\right)}^{T}U\left(ℱ-b\right), \end{array}$$

where *U* ∈ ℝ^*N*×*N*^ is a diagonal matrix denoted as *U* = diag{*u*_1_, *u*_2_,…, *u*_*N*_}. *b* = [*b*_*i*_]_*N*×1_ is an *N*-dimensional indicating vector. The degree matrix *D* is a diagonal matrix with degrees of the node in main diagonal, denoted as *D* = diag{*d*_1_, *d*_2_,…, *d*_*N*_}. Then every pixel is identified uniquely by a node in our undirected graph, where the degree of each vertex is computed as *d*_*i*_ = ∑_*j*_*w*_*ij*_for all the edges that incident on the vertex.*L* is the Laplacian graph matrix, which is denoted as *L* = *D* − *W*. The parameter *α* is a positive constant which controls the tradeoff between two terms.

By the partial derivatives with respect to *ℱ*, the following system of the linear equations is solved for each seed to obtain the probabilities of the unlabeled nodes in the following equation:

(14) $$\begin{array}{c} ℱ={\left(D-W+\lambda U\right)}^{-1}\alpha Ub, \end{array}$$

and after the probabilities of unlabeled nodes are solved, a node *i* can be assigned to the foreground label “+1” if the probability *ℱ*(*i*) ≥ 0. Otherwise, it is assigned to the background label “−1” if the probability *ℱ*(*i*) < 0.

## 4. Experimental Setup and Results

In this section, the experimental results of the improved random walker are shown and the performances are validated on the LIDC dataset. The visual results of extensive experiments and the results of quantitative analysis in terms of overlap score and F-measure are shown. We also validate the sensitivity of the improved random walker by varying the number of background seeds in Section 4.5 and the robustness of running time in Section 4.6, respectively.

Experimental results have demonstrated that the improved random walker is capable of segmenting GGO pulmonary nodules without user interaction. Quantitative and qualitative evaluations on the LIDC dataset also show that the improved random walker significantly improves segmentation performance of GGO pulmonary nodules. All tests are performed on a Windows platform using MATLB R2013a and under the same computer configuration: Intel (R) CPU E3-1225 v5 @3.30 GHz with 4.0 GB RAM.

### 4.1. LIDC Dataset

**Figure 4:**
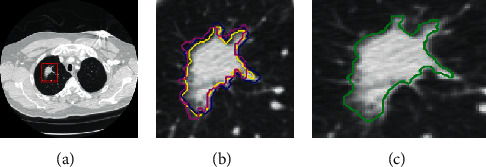
Generating ground truth through the annotations of CT slice. (a) Original CT 2D slice. The pulmonary nodule is displayed with a red rectangle. (b) The outlines of the pulmonary nodule drawn by four radiologists. (c) Ground truth obtained by a 50% consensus criterion.

All experiments are conducted on the LIDC dataset. The Lung Images Dataset Consortium (LIDC) [49] is a web accessible international pulmonary nodule dataset for the evaluation of pulmonary nodule segmentation methods, which contains 1018 CT scans and associated XML files that record the nodule information of a two-phase reading process performed by four board-certified thoracic radiologists. Lung images were acquired by several CT scanners with different manufacturers and pulmonary nodules were judged by four board-certified radiologists.  Figure 4 shows an example result of ground truth generation through the annotations of CT slice. As shown in Figure 4(b), the outlines of the pulmonary nodules were drawn manually by four radiologists. For the visualization purpose, four different colors of the outlines indicate four different segmentation results of the pulmonary nodules obtained by four radiologists. The aquamarine, yellow, blue, and purple colors indicate segmentation results of the pulmonary nodules obtained by four radiologists.  Figure 4(c) shows the corresponding ground truth used in this paper. A 50% consensus criterion [14] is used to produce the outline of ground truth in this paper. We randomly selected 100 CT images with GGO nodules from the LIDC database, which provide different shapes, sizes, and texture information. Their diameters range from 3 mm to 30 mm (average 9.80 mm). All slices used in the experiments are intensity-normalized with gray level from 0 to 255.

### 4.2. Parameter Setting

In the seed acquisition step, there are four parameters that control the location and quantity of the seeds. The parameter *T* is introduced to determine the size of *O*_0_. In this paper, *T* is set a large range from 0.5 to 3.5, which is based on nodule size. One validation metric for segmentation performance is the overlap score, which measures the overlapping area between the segmentation results and ground truth. The overlap score is formulated in the following equation:

(15) $$\begin{array}{c} \text{Overlap score}=\frac{\left|S_{A}\cap S_{M}\right|}{\left|S_{A}\cup S_{M}\right|}, \end{array}$$

where *S*_*A*_ and *S*_*M*_ are the segmentation result and the ground truth, respectively. |*S*_*A*_∩*S*_*M*_| represents the number of pixels in both *S*_*A*_ and *S*_*M*_, and |*S*_*A*_ ∪ *S*_*M*_| is the number of pixels in either *S*_*A*_ or *S*_*M*_, or both. The value of overlap score ranging from 0 to 1 indicates the degree of the accuracy. A high value of overlap score indicates the better segmentation performance. On the contrary, the overlap score has a low value when the segmentation result and the ground truth are inconsistent.

The quantity of background seeds is controlled by the parameter *T*_1_. It is also well known that the random walk method depends on the initial nodule and foreground seeds. We validate how sensitive the improved random walker is to the number of background seeds. First, we run the codes of the proposed method on 10 different cases using the different number of background seeds. The number of background seeds is in the range of [10, 100], where the step length is 20.  Table 1 shows the overlap score results for ten different cases with ten different numbers of background seeds. To better verify the effectiveness of the proposed method, 538 CT images with GGO pulmonary nodules are randomly selected from the LIDC dataset to perform the experiment. Overlap scores slightly increase as the number of background seeds increases. Ultimately, it will remain stable to some extent. The parameter *T*_1_=100 produces considerably good results for the experiments.

**Figure 5:**
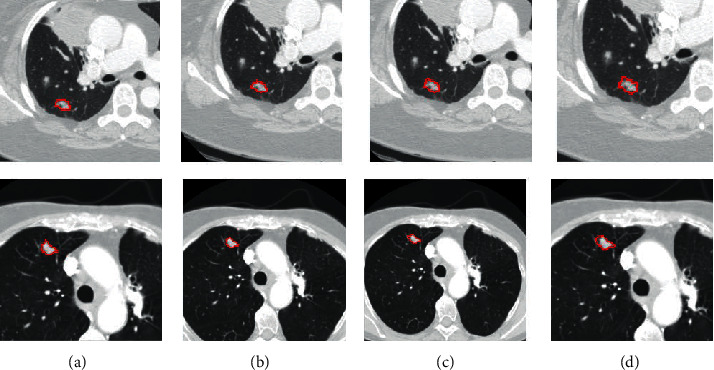
Qualitative comparisons of segmentation results with different values of parameter *α* from 10 to 10^4^. (a)*α*=10. (b)*α*=10^2^. (c)*α*=10^3^. (d)*α*=10^4^.

In the energy function designation step, the parameter *α* in (8) is introduced to control the tradeoff between two terms. When the value of parameter *α* is zero, the segmentation is based on the conventional random walk. The impact of the label constraint term increases as the value of *α* increases. We vary the values of the parameter *α* from 10 to 10^4^ for each CT image to obtain different segmentation results.  Figure 5 shows the segmentation results of the improved random walker with varying parameter *α*. From left to right are the segmentation results obtained with *α*=10, 10^2^, 10^3^, and 10^4^, respectively.  Table 2 shows the overlap scores of 10 cases using four different *α*. As shown in Figure 5 and Table 2, there are not significant changes when *α* is a large positive number. In all experiments, the parameter *α* is set to 10^2^. Similarly, we set *κ*=10 and *η*=5 in the local search strategy process.

### 4.3. Experimental Tests

In this section, the visual analysis of massive experiments is available to validate the improved random walker. The LIDC dataset is used to conduct all experiments. To verify the effectiveness of the acquired nodule seeds, we conduct a comparison experiment between the improved random walker with the acquired nodule seeds and the improved random walker without the acquired nodule seeds. For a fair comparison, the same background seeds are employed in this experiment to reduce the influence of background seeds.

**Figure 6:**
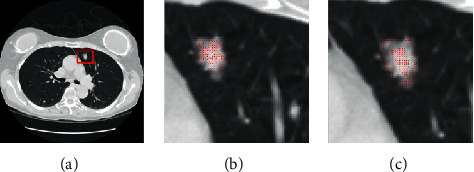
The acquired nodule seeds and improved random walker without the acquired nodule seeds. (a) An original CT lung image. (b) The nodule seeds by user selection. (c) The nodule seeds by geodesic distance and a local search strategy.

**Figure 7:**
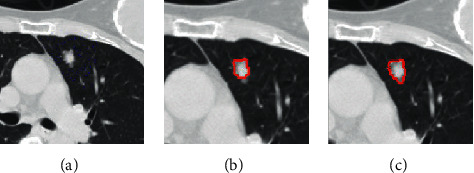
Comparison of segmentation results between improved random walker with the acquired nodule seeds and improved random walker without the acquired nodule seeds. (a) The background seeds by a local search strategy. (b) The segmentation result by the improved random walker without the acquired nodule seeds. (c) The segmentation result of the improved random walker with the acquired nodule seeds.

Figure 6 shows the acquired nodule seeds.  Figure 6(b) shows the nodule seeds obtained by user and Figure 6(c) shows the nodule seeds obtained by geodesic distance and a local search strategy. Red spots specify the nodule seeds.  Figure 7 shows segmentation results by the improved random walker with the acquired nodule seeds and the improved random walker without the acquired nodule seeds.  Figure 7(a) shows the background seeds obtained by a local search strategy. Blue spots specify the background seeds.  Figure 7(b) shows the segmentation results by the improved random walker without the acquired nodule seeds and Figure 7(c) shows the improved random walker with the acquired nodule seeds. As shown in Figure 7(b), many pixels belonging to the nodule region cannot be accurately segmented. From Figure 7(c), we can see that the improved random walker with the acquired nodule seeds improves a GGO pulmonary nodule segmentation. Therefore, the experimental result indicates the benefits of the acquired nodule seeds.

**Figure 8:**
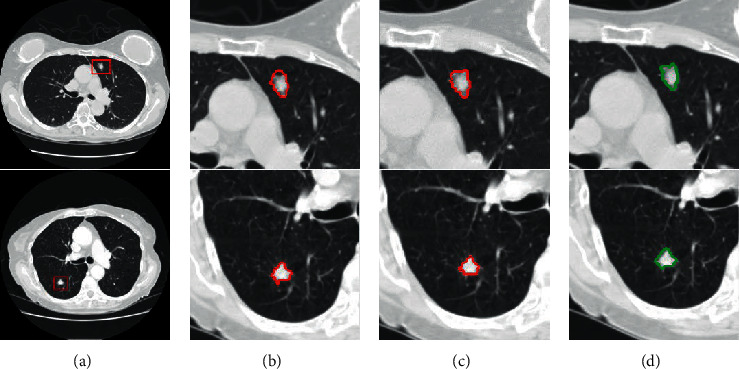
Comparison of segmentation between conventional random walker and improved random walker. (a) Original CT images. (b) The segmentation results by conventional random walker with our seeds. (c) The segmentation results by the improved random walker with our seeds. (d) The ground truth.

To verify the effectiveness of the proposed energy function, we will conduct a quantitative comparison experiment between the improved random walker and the conventional random walk and discuss the comparison results of the conventional random walk. To run random walker, the source codes from the author's homepage were downloaded. The results by the improved random walker are displayed in Figure 8(b). The results by the conventional random walker are displayed in Figure 8(a) for comparison. As shown in Figure 8(a), the conventional random walk yields seriously the oversegmentation phenomenon. It is because conventional random walk has high sensitivity that some nonnodule pixels are labeled as nodule seeds inevitably in the initial nodule seeds acquisition step. In contrast, the improved random walker completely removes the part of oversegmentation. As observed from Figure 8(b), it can be clear that the improved random walker achieves a better segmentation result than the conventional random walk. The outlines of pulmonary nodule segmentation by the improved random walker are more close to the ground truth than conventional random walk, which is shown in Figure 8(d).

Although the conventional random walker can segment out the most part of pulmonary nodules from surrounding pulmonary parenchyma, the pulmonary nodule pixels are more or less leaked into pulmonary parenchyma incorrectly. The improved random walker considers the consistency between the redefined labels and the calculated labels in the new energy function optimization to alleviate the disturbance of seeds to some extent. Therefore, the segmentation performance will be further improved by encouraging the label consistency according to the energy optimization. After adding the label constraint term of the energy function, our method performs well for segmentation of GGO pulmonary nodules, as shown in Figure 8(d). This segmentation improvement may be because the local search strategy of seed acquisition yields the reliable seeds to guide the segmentation.

### 4.4. Quantitative Results

#### 4.4.1. Quantitative Results Using Overlap Score

To verify the effectiveness of the improved random walker, we perform a quantitative comparison between the improved random walker, the conventional random walker with the acquired seeds, and the conventional random walker without the acquired seeds by using the overlap score. 23 CT images with GGO pulmonary nodules are randomly selected from the LIDC dataset, which provide different shapes, sizes, and texture information. In the experiment, the parameter *T* is set to 2, *κ* is set to 10, and *η* is set to 5 in the local search strategy process. The parameter *T*_1_ is set to 100 in background seed acquisition. The parameter *α* is set to 100 in the energy function designation step.

The comparison results of overlap scores are shown in Table 2. The mean value and variance of overlap scores are then calculated in Table 3. The first column shows the case ID numbers. The third column shows the overlap scores calculated by the improved random walker. The fourth and fifth columns show the overlap scores calculated by the conventional random walk with our seeds and the conventional random walk without our seeds, respectively. As shown in Table 3, the improved random walker generated higher average overlap score than the conventional random walk with our seeds, which proves that a label constraint term of energy function can obtain better prior information and further improves the segmentation result. Except for the advantage of the improved energy function process, the comparison between the conventional random walker with our seeds and the conventional random walker without with our seeds also shows the necessity of the acquired seeds. As shown in the fourth and fifth columns of Table 3, the conventional random walk with the acquired seeds obtains the average of 0.8011, and the conventional random walk without the acquired seeds obtains the average of 0.7604. The conventional random walk with the acquired seeds slightly outperforms the conventional random walk without the acquired seeds by less than 0.04 on average. To better verify the effectiveness of the acquired seeds, 538 CT images with GGO pulmonary nodules are randomly selected from the LIDC dataset; the conventional random walk with the acquired seeds obtains the average of 0.8354, and the conventional random walk without the acquired seeds obtains the average of 0.7849. The experimental results show that the acquired seeds are effective.

Hence, the acquired seeds can improve segmentation performance. The improved random walker appears to be more stable, in that it has smaller standard deviation of 0.0529. The results demonstrate that the improved random walker has a higher degree of accuracy in terms of the highest average overlap scores and has a higher degree of robustness in terms of the lowest standard deviations of overlap scores. To better verify the effectiveness of the proposed method, 849 CT images with GGO pulmonary nodules are randomly selected from the LIDC dataset, the proposed method obtains the average of 0.8649, and the conventional random walk obtains the average of 0.7937. The experimental results show that the proposed method outperforms the conventional random walk.

Overall, the improved random walker outperforms the conventional random walk. To validate the effectiveness of the proposed method, an execution time comparison experiment of the proposed method with two other methods was implemented. The conventional random walk without the acquired seeds obtains the average of 3.8497 and the standard deviation of 1.2478. The conventional random walk with the acquired seeds obtains the average of 2.6462 and the standard deviation of 0.8394. The improved random walker obtains the smaller average of 1.3536 and the standard deviation of 0.1165. The results demonstrate that the improved random walker has a smaller execution time.

#### 4.4.2. Quantitative Results Using F-Measure

To further verify the performance of the improved random walker, we adopt the second metric, F-measure. Precision is defined as the ratio of the sum of intensities inside the nodule region to the total intensities calculated in the CT imaging. Recall is defined as the ratio of the total pixels captured inside nodule region to the area of the user annotated window. F-measure [51] is defined as the weighted harmonic mean between the Precision and Recall values, which is formulated as follows:

(16) $$\begin{array}{c} F-{\text{measure}}_{\beta }=\frac{\left(1+\beta \right)\cdot \text{Precision}\cdot \text{Recall}}{\left(\beta \cdot \text{Precision}+\text{Recall}\right)}, \end{array}$$

where *β* is a tradeoff factor controlling the importance of Precision and Recall. In our experiments, it is fixed to 0.3 empirically to weight Precision more than Recall.

In this experiment, 10 cases with GGO pulmonary nodules are randomly selected from the LIDC dataset, which provide different shapes, sizes, and texture information. For each binary map of pulmonary nodules, the Precision, Recall, and F-measure are calculated on ten different images. The results are shown in Table 4. As shown in Table 4, the improved random walker obtains a high average F-measure of 0.8951. To evaluate the proposed method's efficiency, the comparison is produced between the improved random walker and the other two methods by F-measure. The results are shown in Table 5. As shown in Table 5, the improved random walker obtains a high average F-measure of 0.8951.

Further, to evaluate the computational efficiency, the comparison between the improved random walker and Kubota's method [14] in terms of the executive times is shown in Table 6, which is measured in seconds on LIDC dataset. The improved random walker required 1.35 seconds to segment each CT image on average, which was much faster than Kubota's method [14]. We expect that the execution time would be better.

### 4.5. Comparisons with Other State-of-the-Art Methods

We evaluate the improved random walker with several state-of-the-art methods, including Kostis's method [3], Okada's method [23], Kuhnigk's method [20], Kostis's method [14], Messay's method [52], Ye's method [33], and Wang's method [34]. These methods employed the LIDC database to evaluate the performance of pulmonary nodules segmentation and the overlap scores have been calculated by the authors. For the evaluation of nodule segmentation methods from the LIDC dataset, deep learning network can obtain satisfactory segmentation results, but it relies more on the data to train the network. They cannot be guaranteed using the same cases. Therefore, the evaluation results may have the variability for different nodules to a certain degree. Despite these differences, the performance comparison between the improved random walker and the state-of-the-art methods is valuable.  Table 7 summarizes the average and standard deviation of overlap score values of five segmenting methods on the LIDC dataset. In total, we obtained the average overlap score of 0.86. As we can see, Okada's method [23] presented an overlap score of 0.45 ± 0.21, which is relatively lower compared to other methods due to the discrepancy between the ellipsoid model and nonellipsoidal nodules, which did not well handle nonellipsoidal GGO nodules. Kostis's method [3] and Kuhnigk's [20] method presented the overlap scores of 0.57 ± 0.20 and 0.56 ± 0.18, respectively. Kostis's method potentially assumed that pulmonary nodule is (usually) roughly spherical or ellipsoidal shapes. So, it also did not well handle GGO nodules. Kuhnigk's method used morphological opening processing for pulmonary nodule segmentation, which is suitable for both small and regular nodules. However, for GGO nodules with fuzzy and irregular boundary, the segmentation results were unsatisfactory, since erosion operation may remove a portion of the nodule. Therefore, this method presented a relatively lower overlap score. Kubota's method [14] reported the overlap score of 0.66 ± 0.18, which has a relatively high overlap score compared to the above three methods. Kubota et al. employed competition-diffusion (CD) method to obtain the foreground object and region growing to obtain final segmentation results, which was also less robust and accurate, resulting in undersegmentation results for GGO nodules compared to our proposed method, especially for GGO nodule with spiculations. Messay et al. [52] reported the overlap score of 0.77 ± 0.09 for the hybrid method, which obtained a higher value than those of the above-mentioned methods. The performance of segmentation has a considerable boost by using a regression neural network approach; however, this method required carefully manual supplied control points to improve the segmentation results. Ye's method [33] was used in AlexNet and GoogLeNet to detect GGO pulmonary nodules and created the input image of the three-dimensional features to train the deep network, which obtained the overlap score of 0.81 ± 0.13. Ye's method has a relatively higher overlap score than the above-mentioned methods. Wang's method [34] built a cascade architecture with both segmentation and classification networks for automatic GGO nodules segmentation and obtained the overlap score of 0.84 ± 0.10. The cascade model in the data level performs better and is more stable than Ye's method. Herein, we achieved a relatively higher overlap score compared to other methods, 0.86 ± 0.08, which is a boost in comparison to the other methods. The good performance of the proposed method can be attributed to the powerful discriminating affinity matrix and a label constraint term of the energy function for handling the fuzzy and irregular GGO nodules. In addition, the rapid training of large amounts of data and the determination of hyperparameters are also problems to be solved in the future of deep learning. The existing network model or network model combined with traditional methods will become a popular trend.

## 5. Discussion

The irregular shapes, fuzzy boundaries, and low contrasts between the pulmonary nodule and surrounding background prohibit accurate GGO pulmonary nodule segmentation using simple methods based on thresholding, region grow, and morphological methods. It is clear that the segmentation of GGO pulmonary nodules requires a specialized method. The random walker has been paid more and more attention for interactive image segmentation. It produces a good segmentation. The intensity, texture, and spatial features are incorporated to construct a new affinity matrix. It strengthens discriminative power between two adjacent nodes on the graph. To automatically acquire seeds, the geodesic distance is introduced and a novel local search strategy is presented to automatically select reliable seeds. For segmentation, a label constraint term is introduced to the energy function of original random walker, which alleviates the accumulation of errors caused by the initial seeds acquisition. The improved random walker requires no user interaction.

The differences in performance of the improved random walker and the conventional random walker were found to be statistically significant in terms of the overlap score. Based on 23 cases consisting of different sizes, shapes, and locations of pulmonary nodules, the improved random walker method obtains a higher average overlap score compared to the conventional random walker, which is shown in Table 3. This good segmentation performance can probably be ascribed to the acquired seeds and the energy function designation. To further verify the performance of the improved random walker, we adopt the second metric, F-measure. The improved random walker has a high average and a low standard deviation of F-measure values. Dakua and Sahambi [53] proposed a method of the automatic seed selection using cantilever beam equation and a combined adaptive threshold technique and located the seeds on demand at different locations around LV boundary. The proposed method adopted the adaptive threshold method to roughly separate the nodule from the background. The pixels are roughly identified as a part of pulmonary nodule, whose intensities are greater compared to the global threshold. The remaining pixels are considered as the background pixels. Geodesic distance was adopted to create a small region from an initial segmentation result. To acquire the nodule seeds as uniformly as possible, a local search strategy was introduced to acquire the other nodule seeds.

## 6. Conclusions and Future Works

In this paper, we propose an improved random walker method for segmentation of GGO pulmonary nodules. This algorithm is an extension of the previously proposed algorithm [54]. The automatic seeds acquisition is significant when a massive CT dataset needs to be examined. The geodesic distance and a local search strategy are introduced to automatically acquire GGO nodule and background seeds. The main advantage of the improved random walker is to automatically and accurately segment GGO pulmonary nodules without any user interaction and shape assumption. The proposed local search strategy incorporates intensity and texture features to define a similarity rule, which can assign the pixels to a nodule seed set or a background seed set. The proposed affinity matrix consists of an adjacency component and a feature-based component. The adjacency component is defined based on the spatial distance. The feature-based component is defined based on intensity and texture features. The proposed energy function adds a label constraint term, which alleviates the accumulation of errors caused by the initial seeds acquisition. The weight of the label constraint term is based on fuzzy membership value, which is calculated by building two GMM models. The proposed method is implemented efficiently and simply.

The results have demonstrated the robustness and efficiency of the proposed segmentation method for the segmentation of GGO pulmonary nodules. The proposed energy function is minimized by solving a linear system. The experimental results have shown that the improved random walker achieves satisfactory segmentation results by both quantitative and qualitative performance assessment, especially in complex GGO pulmonary nodules.

In future work, we will improve the accuracy and efficiency of the improved random walker for some complex GGO nodules. We also will extend this method to segment various pulmonary nodules. When we directly calculate the inverse matrix in (14), the computation cost is expensive, especially when the number of image pixels is very large. Therefore, we will research how to speed up the computation of the improved random walker.

## Figures and Tables

**Table 1:** Overlap scores of 10 cases using ten different numbers of background seeds.

ID	20	40	60	80	100
1	0.8426	0.8675	0.8749	0.9057	0.9159
2	0 9058	0.9058	0.9103	0.9134	0.9147
3	0.8029	0.8574	0.8584	0.8828	0.8925
4	0 8142	0.8182	0.8448	0.8645	0.8828
5	0.0059	0.0059	0.8043	0.8429	0.9050
6	0.8067	0.8067	0.8352.	0.8578	0.8638
7	0.0034	0.0034	0 7952	0.8049	0.8472
8	0.8829	0.8879	0.9042	0.9089	0.9121
9	0.0074	0.0198	0.8520	0.8743	0.8829
10	0.8142	0.8488	0.8649	0.9024	0.9112

**Table 2:** Overlap scores of 10 cases using four different *α*.

ID	10	10^2^	10^3^	10^4^
1	0.8468	0.8642	0.8437	0.8429
2	0.9215	0.9430	0.9104	0.9175
3	0.8522	0.8846	0.8577	0.8782
4	0.9369	0.9642	0.9013	0.9040
5	0.8434	0.8658	0.8529	0.8514
6	0.9400	0.9708	0.9230	0.9255
7	0.9061	0.9391	0.9116	0.9164
8	0.8849	0.9072	0.9042	0.9022
9	0.9216	0.9300	0.9253	0.9108
10	0.9242	0.9588	0.9562	0.9424

**Table 3:** Overlap scores of 23 CT images obtained using the improved random walker and the conventional random walk. See the text for more details.

ID	The number of seeds	The improved RW	RW with our seeds	RW without our seeds	Times
1	276	0.9193	0.7727	0.7452	1.2500
2	235	0.8903	0.9291	0.8434	1.2650
3	297	0.9203	0.8024	0.7634	1.2521
4	349	0.8231	0.7054	0.6901	1.3190
5	216	0.9321	0.9193	0.9025	1.4760
6	197	0.8929	0.7544	0.7146	1.3440
7	302	0.8340	0.7053	0.7568	1.3280
8	189	0.8853	0.7619	0.7023	1.6310
9	243	0.8727	0.8820	0.7823	1.3440
10	379	0.9341	0.8319	0.8726	1.3260
11	197	0.8290	0.8920	0.8340	1.6950
12	264	0.8631	0.8631	0.7920	1.2810
13	375	0.8498	0.8012	0.7034	1.3520
14	294	0.8920	0.8290	0.8401	1.2970
15	176	0.9352	0.8031	0.7432	1.4230
16	276	0.8860	0.8460	0.6931	1.4690
17	307	0.7964	0.7254	0.7350	1.3130
18	238	0.9453	0.8015	0.7031	1.4220
19	398	0.8624	0.8106	0.8326	1.2190
20	289	0.9540	0.8342	0.7920	1.2820
21	208	0.7786	0.7326	0.7021	1.3550
22	178	0.7695	0.7690	0.7431	1.3130
23	267	0.8442	0.6524	0.6014	1.2340
Mean std.		0.8743	0.8011	0.7604	1.3561
		0.0529	0.0708	0.0710	0.1165

**Table 4:** Quantitative results of 10 cases using F-measure.

ID	CT scan	Precision	Recall	F-measure
1	LIDC-IDRI-0260	0.9805	0.8653	0.9513
2	LIDC-IDRI-0195	0.9509	0.8369	0.9219
3	LIDC-IDRI-0060	0.9847	0.6898	0.8963
4	LIDC-IDRI-0045	0.9624	0.8209	0.9256
5	LIDC-IDRI-0044	0.9850	0.8845	0.9599
6	LIDC-IDRI-0003	0.9521	0.8407	0.9238
7	LIDC-IDRI-0186	0.9899	0.4624	0.7837
8	LIDC-IDRI-0087	0.8487	0.8197	0.8418
9	LIDC-IDRI-0884	0.9403	0.9214	0.9359
10	LIDC-IDRI-0052	0.8395	0.7260	0.8103
Mean std.		0.9434	0.7868	0.8951
		0.0550	0.1333	0.0614

**Table 5:** Quantitative results of 10 cases using F-measure by the proposed method and the other two methods.

ID	CT scan	RW without our seeds	RW with our seeds	Proposed method
1	LIDC-IDRI-0260	0.9202	0.9314	0.9513
2	LIDC-IDRI-0195	0.9146	0.9279	0.9219
3	LIDC-IDRI-0060	0.8249	0.8435	0.8963
4	LIDC-IDRI-0045	0.8986	0.9014	0.9256
5	LIDC-IDRI-0044	0.9146	0.9271	0.9599
6	LIDC-IDRI-0003	0.8762	0.8920	0.9238
7	LIDC-IDRI-0186	0.6879	0.7249	0.7837
8	LIDC-IDRI-0087	0.7858	0.8171	0.8418
9	LIDC-IDRI-0884	0.8632	0.8969	0.9359
10	LIDC-IDRI-0052	0.7868	0.7935	0.8103
Mean std.		0.8473	0.8656	0.8951
		0.0756	0.0690	0.0614

**Table 6:** Comparison of execution times of the improved random walker and Kubota's method.

Method	Time (s)
Kubota's method [14]	3.82 ± 4.91
Our method	1.35 ± 0.12

**Table 7:** Comparison results of overlap scores on LIDC dataset in the state-of-the-art methods.

Method	Overlap scores
Kostis et al. (2003) [3]	0.57 ± 0.20
Okada et al. (2005) [23]	0.45 ± 0.21
Kuhnigk et al. (2006) [20]	0.56 ± 0.18
Kubota et al. (2011) [14]	0.66 ± 0.18
Messay et al. (2015) [52]	0.77 ± 0.09
Ye et al. (2019) [33]	0.81 ± 0.13
Wang et al. (2021) [34]	0.84 ± 0.10
Our method	0.86 ± 0.08

## Data Availability

Supporting data are available from LIDC database:https://download.csdn.net/download/aristocles118/12390876.

## References

[1] Girvin F., Ko J. P. (2008). Pulmonary nodules: detection, assessment, and CAD. *American Journal of Roentgenology*.

[2] Kawata Y., Nakaoka M., Niki N., Ohmatsu H. (2006). Growth-rate estimation of pulmonary nodules in three-dimensional thoracic CT images based on CT density histogram analysis and its application to nodule classification. *Medical Imaging*.

[3] Kostis W. J., Reeves A. P., Yankelevitz D. F., Henschke C. I. (2003). Three-dimensional segmentation and growth-rate estimation of small pulmonary nodules in helical CT images. *IEEE Transactions on Medical Imaging*.

[4] Reeves A. P., Chan A. B., Yankelevitz D. F., Henschke C. I., Kressler B., Kostis W. J. (2006). On measuring the change in size of pulmonary nodules. *IEEE Transactions on Medical Imaging*.

[5] Pedersen J. H., Saghir Z., Wille M., Thomsen L. H., Skov G. B., Ashraf H. (2016). Ground-glass opacity lung nodules in the era of lung cancer CT screening: radiology, pathology, and clinical management. *Oncology*.

[6] Grady L. (2006). Random walks for image segmentation. *IEEE Transactions on Pattern Analysis and Machine Intelligence*.

[7] Tan J. H., Acharya U. R., Lim C. M., Abraham K. T. (2013). An interactive lung field segmentation scheme with automated capability. *Digital Signal Processing*.

[8] Patz T., Preusser T. (2012). Segmentation of stochastic images with a stochastic random walker method. *IEEE Transactions on Image Processing*.

[9] Fabijanska A., Goclawski J. (2015). The segmentation of 3D images using the random walking technique on a randomly created image adjacency graph. *IEEE Transactions on Image Processing*.

[10] Eslami A., Karamalis A., Katouzian A., Navab N. (2013). Segmentation by retrieval with guided random walks: application to left ventricle segmentation in MRI. *Medical Image Analysis*.

[11] Mi H., Petitjean C., Vera P., Ruan S. (2015). Joint tumor growth prediction and tumor segmentation on therapeutic follow-up PET images. *Medical Image Analysis*.

[12] Armato S. G., Giger M. L., MacMahon H. (2016). A novel approach to CAD system for the detection of lung nodules in CT images. *Computer Methods and Programs in Biomedicine*.

[13] Messay T., Hardie R. C., Rogers S. K. (2010). A new computationally efficient CAD system for pulmonary nodule detection in CT imagery. *Medical Image Analysis*.

[14] Kubota T., Jerebko A. K., Dewan M., Salganicoff M., Krishnan A. (2011). Segmentation of pulmonary nodules of various densities with morphological approaches and convexity models. *Medical Image Analysis*.

[15] Li Q., Li F., Doi K. (2008). Computerized detection of lung nodules in thin-section CT images by use of selective enhancement filters and an automated rule-based classifier. *Academic Radiology*.

[16] Dehmeshi J., Amin H., Valdivieso M., Ye X. J. (2008). Segmentation of pulmonary nodules in thoracic CT scans: a region growing approach. *IEEE Transactions on Medical Imaging*.

[17] Liu H., Zhang C. M., Su Z. Y., Wang K., Deng K. (2015). Research on a pulmonary nodule segmentation method combining fast self-adaptive FCM and classification. *Computational and Mathematical Methods in Medicine*.

[18] Sivakumar S., Chandrasekar C. (2020). Kernel-based Bayesian clustering of computed tomography images for lung nodule segmentation. *IET Image Processing*.

[19] Badura P., Pietka E. (2014). Soft computing approach to 3D lung nodule segmentation in CT. *Computers in Biology and Medicine*.

[20] Kuhnigk J. M., Dicken V., Bornemann L. (2006). Morphological segmentation and partial volume analysis for volumetry of solid pulmonary lesions in thoracic CT scans. *IEEE Transactions on Medical Imaging*.

[21] Aoyama M., Li Q., Katsuragawa S., Li F., Sone S., Doi K. (2003). Computerized scheme for determination of the likelihood measure of malignancy for pulmonary nodules on low-dose CT images. *Medical Physics*.

[22] Lee Y., Hara T., Fujita H., Itoh S., Ishigaki T. (2001). Automated detection of pulmonary nodules in helical CT images based on an improved template-matching technique. *IEEE Transactions on Medical Imaging*.

[23] Okada K., Comaniciu D., Krishnan A. (2005). Robust anisotropic Gaussian fitting for volumetric characterization of Pulmonary nodules in multislice CT. *IEEE Transactions on Medical Imaging*.

[24] Cheng H. L., Jung S. J. (2020). Automatic segmentation for pulmonary nodules in CT images based on multifractal analysis. *IET image processing, nol*.

[25] Chen K., Li B., Tian L. F., Zhu W. B., Bao Y. H. (2014). Vessel attachment nodule segmentation using integrated active contour model based on fuzzy speed function and shape-intensity joint Bhattacharya distance. *Signal Processing*.

[26] Farag A. A., Munim H., Graham J. H., Farag A. A. (2013). A novel approach for lung nodules segmentation in chest CT using level sets. *IEEE Transactions on Image Processing*.

[27] Li B., Chen Q. L., Peng G. M. (2016). Segmentation of pulmonary nodules using adaptive local region energy with probability density function-based similarity distance and multi-features clustering. *BioMedical Engineering Online*.

[28] Wang S., Zhou M., Liu Z. (2017). Central focused convolutional neural networks: developing a data-driven model for lung nodule segmentation. *Medical Image Analysis*.

[29] Yan H. L., Lu H. J., Ye M. C., Yan K., Xu Y., Jin Q. Improved mask R-CNN for lung nodule segmentation.

[30] Wang W. Z., Feng R. W., Chen J. T., Yu H., Chen D. Z., Wu J. (2019). Nodule-plus R-CNN and deep self-paced active learning for 3D instance segmentation of pulmonary nodules. *IEEE Access*.

[31] Liu H., Cao H. C., Song E. M. (2019). A cascaded dual-pathway residual network for lung nodule segmentation in CT images. *Physica Medica*.

[32] Cao H. C., Liu H., Song E. M. (2020). Dual-branch residual network for lung nodule segmentation. *Applied Soft Computing*.

[33] Ye W., Gu W W., Guo X. (2019). Detection of pulmonary ground-glass opacity based on deep learning computer artificial intelligence. *BioMedical Engineering Online*.

[34] Wang D., Zhang T., Li M., Bueno R., Jayendaer J. (2020). 3D deep learning based classification of pulmonary ground glass opacity nodules with automatic segmentation. *Computerized Medical Imaging and Graphics*.

[35] Huang X., Sun W. Q., Tseng T. L., Li C. Q., Qian W. (2019). Fast and fully-automated detection and segmentation of pulmonary nodules in thoracic CT scans using deep convolutional neural networks. *Computerized Medical Imaging and Graphics*.

[36] Diciotti S., Lombardo S., Falchini M., Picozzi G., Mascalchi M. (2011). Automated segmentation refinement of small lung nodules in CT scans by local shape analysis. *IEEE Transactions on Biomedical Engineering*.

[37] Murphy A. K., Ginneken B. V., Schilham A. M. R., Hoop B. J. D., Gietema H. A., Prokop M. (2009). A large-scale evaluation of automatic pulmonary nodule detection in chest CT using local image features and k-nearest-neighbour classification. *Medical Image Analysis*.

[38] Lin P. L., Huang P. W., Lee C. H., Wu M. T. (2013). Automatic classification for solitary pulmonary nodule in CT image by fractal analysis based on fractional Brownian motion model. *Pattern Recognition*.

[39] Han F. F., Wang H. F., Zhang G. P. (2015). Texture feature analysis for computer-aided diagnosis on pulmonary nodules. *Journal of Digital Imaging*.

[40] Park S., Kim B., Lee J., Goo J. M., Shin Y. G. (2011). GGO nodule volume-preserving nonrigid lung registration using GLCM texture analysis. *IEEE Transactions on Biomedical Engineering*.

[41] Leung T., Malik J. (2001). Representing and recognizing the visual appearance of materials using three-dimensional textons. *International Journal of Computer Vision*.

[42] Ning J. F., Zhang L., Zhang D., Wu C. K. (2010). Interactive image segmentation by maximal similarity based region merging. *Pattern Recognition*.

[43] Dhara A. K., Mukhopadhyay S., Dutta A., Garg M., Khandelwal N. (2016). A combination of shape and texture features for classification of pulmonary nodules in lung CT images. *Journal of Digital Imaging*.

[44] Manikandan T., Bharathi N. (2016). Lung cancer detection using fuzzy auto-seed cluster means morphological segmentation and SVM classifier. *Journal of Medical Systems*.

[45] Liu L., Lao S., Fieguth P. W., Guo Y., Wang X., Pietikäinen M. (2016). Median robust extended local binary pattern for texture classification. *IEEE Transactions on Image Processing*.

[46] Rastghalam R., Pourghassem H. (2016). Breast cancer detection using MRF-based probable texture feature and decision-level fusion-based classification using HMM on thermography images. *Pattern Recognition*.

[47] Guo Z., Wang X., Zhou J., You J. (2016). Robust texture image representation by scale selective local binary patterns. *IEEE Transactions on Image Processing*.

[48] Bianconi F., Fernandez A. (2007). Evaluation of the effects of Gabor filter parameters on texture classification. *Pattern Recognition*.

[49] Armato S. G., McLennan G., Bidaut L., Mcnitt-Gray M. F. (2011). The lung image database Consortium, (LIDC) and image database resource initiative (idri): a completed reference database of lung nodules on CT scans. *Medical Physics*.

[50] Jacobs C., Rikxoort E. V., Twellmann T. (2014). Automatic detection of subsolid pulmonary nodules in thoracic computed tomography images. *Medical Image Analysis*.

[51] Zou K. H., Warfield S. K., Aditya B. (2004). Statistical validation of image segmentation quality based on a spatial overlap index. *Academic Radiology*.

[52] Messay T., Hardie R. C., Tuinstra T. R. (2015). Segmentation of pulmonary nodules in computed tomography using a regression neural network approach and its application to the Lung Image Database Consortium and Image Database Resource Initiative dataset. *Medical Image Analysis*.

[53] Dakua S. P., Sahambi J. S. (2010). Automatic left ventricular contour extraction from cardiac magnetic resonance images using cantilever beam and random walk approach. *Cardiovascular Engineering*.

[54] Li X. X., Liu F. (2019). Random walks with GMM statistical inference algorithm for segmentation of ground glass opacity pulmonary nodules. *12th International Symposium on Computational Intelligence and Design (ISCID)*.

